# Scene memory and spatial inhibition in visual search: A neural dynamic process model and new experimental evidence

**DOI:** 10.3758/s13414-019-01898-y

**Published:** 2020-02-11

**Authors:** Raul Grieben, Jan Tekülve, Stephan K. U. Zibner, Jonas Lins, Sebastian Schneegans, Gregor Schöner

**Affiliations:** 1 Institut für Neuroinformatik, Ruhr-Universität Bochum, Universitätsstraße 150, 44780 Bochum, Germany; 2 Department of Psychology, University of Cambridge, Cambridge, CB2 3EB UK

**Keywords:** Visual search, Visual working memory, Neural network modeling

## Abstract

Any object-oriented action requires that the object be first brought into the attentional foreground, often through visual search. Outside the laboratory, this would always take place in the presence of a scene representation acquired from ongoing visual exploration. The interaction of scene memory with visual search is still not completely understood. Feature integration theory (FIT) has shaped both research on visual search, emphasizing the scaling of search times with set size when searches entail feature conjunctions, and research on visual working memory through the change detection paradigm. Despite its neural motivation, there is no consistently neural process account of FIT in both its dimensions. We propose such an account that integrates (1) visual exploration and the building of scene memory, (2) the attentional detection of visual transients and the extraction of search cues, and (3) visual search itself. The model uses dynamic field theory in which networks of neural dynamic populations supporting stable activation states are coupled to generate sequences of processing steps. The neural architecture accounts for basic findings in visual search and proposes a concrete mechanism for the integration of working memory into the search process. In a behavioral experiment, we address the long-standing question of whether both the overall speed and the efficiency of visual search can be improved by scene memory. We find both effects and provide model fits of the behavioral results. In a second experiment, we show that the increase in efficiency is fragile, and trace that fragility to the resetting of spatial working memory.

## Introduction

Bringing an object into the attentional foreground is the first step of most intentional actions that are directed at the outer world (Tatler & Land, 2016). Attentional selection is also central to many communicative acts, both when we direct speech at another human to refer to an object in the environment and when we as a listener perceptually ground a speech act of another human (Richter et al., 2017). This attentional selection of an object typically requires visual search if the visual scene is novel or constantly changing, although under natural conditions, it will often be aided by scene understanding and scene memory (Hollingworth, 2009; Võ & Henderson, 2010, 2012; Hollingworth, 2012b; for reviews, see Võ & Wolfe, 2015, Hollingworth, 2012a).

Visual search has been intensely studied in experimental psychology (for reviews, see Folk, 2015; Wolfe, 1998, 2015, 2017, 2018). In laboratory settings, the visual features that are assumed to guide visual search are carefully controlled. Since Anne Treisman’s seminal work on feature integration theory (Treisman & Gelade, 1980), the question how visual search is guided by individual or combinations of feature dimensions has been a dominant theme of that research (Wolfe & Horowitz, 2017). In particular, it has been intensely studied how the amount of time needed to find a cued object scales with the number of distractor items, or with the metric differences between targets and distractors, and the findings have been used to diagnose the underlying process organization (Duncan & Humphrey, 1989; Friedman-Hill & Wolfe, 1995; Wolfe, 1998, 2014).

In the classical picture (Treisman, 1998), a strong increase of the search time with the number of distractors is indicative of the sequential selection of spatial locations to probe the match between target and attended object individually. According to feature integration theory, the need for sequential processing of objects arises because attentional selection is necessary to bind the features (such as color, shape, and orientation) at a single location together. This sequential processing is therefore a signature of conjunctive search tasks, in which the target is defined by a combination of multiple features. In contrast, weak or absent increase of search time with the number of distractors is indicative of processes operating in parallel on spatially distributed locations. Such highly effective searches are possible, for instance, in search guided by a single feature dimension. Modern perspectives are more nuanced as to where the constraints on visual search come from, but continue to emphasize this observable characteristic (for reviews, see Carrasco 2011; Wolfe & Horowitz^2017^).

Feature integration theory has also been applied to the study of visual working memory, and has been extended to the object file theory (Kahneman et al., 1992). The same basic principle—that selective attention is required to bind different visual features of an object together—is here applied to explain how memory representations of a visual scene are formed, and how limitations in working memory arise. Again, the processing demands of feature conjunctions led to a number of experimentally observable signatures in probes of visual (or scene) working memory consistent with feature integration theory (Wheeler & Treisman, 2002; Treisman & Zhang, 2006). Feature integration theory therefore provides a theoretical foundation for the link between visual search and visual working memory. In recent years, a drive to understanding visual search under natural conditions has brought the role of memory and knowledge in visual search more strongly into the foreground (Hollingworth, 2012a). Yet, that role remains controversial, as reviewed below.

### Neural process accounts of feature integration theory

Although feature integration theory was framed in neural terms, invoking cortical feature maps over space as basic format for visual representations, there is to this day no formalized neural process account of the theory. Feature integration theory itself, as a verbal theory, invoked concepts of information processing when it talked about object files that are created, stored, and accessed. Formalized theories of visual search were built within the fold of mathematical psychology. Guided visual search is perhaps the theory of visual search that has the broadest reach and has been most thoroughly tested against experiments (Wolfe, 2007). Guided visual search postulates that an early parallel stage of search is followed by a serial examination of candidate items (Wolfe, 2007).

At the core of guided visual search is an information processing algorithm that starts a diffusion process for each examined item to decide its match to the search criteria. Once the decision has been made, the diffusion is reset and can be restarted for the next item. Competitive guided search (Moran et al., 2013) adds neural mechanisms into the selection process by introducing mutual inhibition as a mechanism, but retains the information processing core.

An alternative formalization is attentional engagement theory which recognizes that metric differences among distractors and between targets and distractors matter (Duncan & Humphrey, 1989). This account has been implemented in a connectionist architecture (Humphreys & Müller, 1993), in which inhibitory and excitatory coupling among feature encoding units leads to grouping effects that explain how search for feature conjunctions can occur pre-attentively (Humphreys, 2016). Heinke and colleagues (Heinke & Humphreys, 2003, 2011; Abadi et al.,, 2019) have proposed neural models of visual attention that make use of visual templates to represent known objects and to determine their match to stimuli in the visual array. Strictly speaking, these models are an alternative to rather than an implementation of feature integration theory. In these models, spatial selection emerges from a neural network that gates projections from all visual locations to a neural representation of the focus of attention. Similar ideas have been used by some of us to model object recognition (Lomp et al., 2017). We will examine the functional role of this framing of visual search in the Discussion. The link of visual search to scene memory is not a topic in this class of models.

There are accounts for visual search that are neurally mechanistic at a lower level of description (Deco & Rolls, 2004). Their capacity to capture the behavioral signatures of conjunctive search is much less developed. Closest to what we aim for in this article are accounts that are formulated in the same theoretical framework of neural dynamics, such as Hamker(2005, 2006) and Fix et al., (2011). These are based on the influential concept of salience maps for visual attention and search (Itti and Koch, 2000), but extend them by neurally plausible mechanisms for top-down modulation and sequential processing of visual arrays. Chikkerur et al., (2010) proposed a graphical model of visual attention that provides an integrated formal account for feature binding in terms of probabilistic inference. However, the deployment of spatial attention to specific locations remains outside the Bayesian framework. None of these accounts addresses both visual search and its interaction with visual working memory.

Our first goal in this article is to provide a complete neural process account for the interaction between visual search and visual working memory. We use a scenario in which human observers are exposed to a visual scene, and are cued by a sample object that appears abruptly. Visual search is enacted by pointing at a matching object’s spatial location. We provide a neural process account that integrates the three core components of visual orientation to objects in the environment: (1) Visual exploration that builds a scene working memory; (2) visual attention directed to locations of visual transients and extraction of the visual features at that location; and (3) visual search for matching objects.

A neural process account is characterized, we stipulate, by complete autonomy. At the level of description of neural population activation (Erlhagen et al., 1999; Purushothaman and Bradley, 2005), autonomy amounts to the continuous evolution in time of activation patterns driven entirely by sensory inputs and recurrent neural connectivity or interaction. Within the framework of dynamic field theory (Schöner et al., 2016), detection and selection decisions emerge from instabilities of the neural dynamics of such populations. Sequences of such decisions emerge from the interactions within a neural dynamic network of populations, that forms a neural dynamic architecture. Thus, the neural processes in this framework fundamentally evolve in parallel across the entire architecture, while sequential processing steps emerge under the right conditions. A demonstration of autonomy in this sense consists of driving a neural dynamic architecture by real, online sensory input from a vision sensor, here a video camera, and generating outcomes as stable patterns of neural activation that can be acted out.

Our account builds on earlier work by Schneegans et al., (2016), in which we established a neural dynamic architecture that autonomously builds a scene working memory, which can then be probed in a change detection paradigm (Wheeler and Treisman, 2002). We demonstrated the differences between detection of change along a single feature dimension as compared to change of feature conjunctions. The special role of space as a feature dimension was demonstrated by comparing change detection with and without shuffling of spatial locations of objects (Treisman & Zhang, 2006). However, the model has not been used to quantitatively fit behavioral results. In this article, we retain some of the key elements of that earlier model and expand it by functional capacities to detect and retain a visual cue and to perform visual search for the cued object. We build in many respects on the overall architecture of guided visual search (Wolfe, 2007).

### Visual search and memory

Most theories of visual search acknowledge that visual search is guided by a search template stored in memory (Duncan & Humphrey, 1989; Bundesen, 1990; Wolfe, 2007). If the target remains the same across trials, the search template is stored in long-term memory (LTM). Otherwise, a search template is stored in visual working memory (VWM: Woodman et al.,, 2007) on each trial. There is strong evidence that the content of working memory (WM) guides visual search (Soto et al.,, 2005, for a review, see Soto et al.,, 2008). Both spatial and non-spatial working memory may play a role in inefficient visual search as supported by the observation of considerable overlap of the recruited cortical networks (Anderson et al., 2010).

Understanding how the neural processes of visual search and of the construction and maintenance of scene memory are integrated is a theoretical challenge. This has been the focus of recent empirical research. The simplest question is if scene memory improves and accelerates inefficient visual search. This question has been explored in a variety of paradigms. Repeated search experiments have provided clear evidence that memory may reduce the time needed to find the cued item. Wolfe and others have argued, however, that this reduction is not indicative of an improved efficiency of visual search itself. Efficiency is estimated from how search times scale with the number of distractor items, and no improvement of efficiency with prior exposure to the scene has been found (Wolfe et al.,, 2000, 2002; Kunar et al., 2008). The reduction of response time may thus reflect primarily facilitation of the pre- and post-search components of visual attention.

Becker and Pashler (2005) have similarly found that a preview of the scene did not decrease the slopes of reaction time against set size functions. Overall, reaction times were shortened by preview up to a capacity limit of three items. Becker and Pashler (2005) argued that observers were able to retain the featural identity of up to three items during preview, but did not profit from preview for items whose identities had not been retained.

If a searched item is already actively held in scene memory, then its attentional selection should be almost immediate and should not be affected by the number of other objects in the visual array. A simple reason why such a strong improvement of visual search efficiency is not readily observable is, of course, the limited capacity of working memory. As the number of items in the visual array is increased, the probability that the searched item can be successfully retrieved from working memory drops strongly, consistent with classical views of only 3–4 slots to retain items in memory (Luck & Vogel, 1997) or alternative accounts of a continuous, but limited memory resource (Ma et al., 2014).

Improved efficiency would thus be limited to a small portion of the scaling law, which is evaluated for efficiency at much larger numbers of items. Specifically, the slope of reaction time as a function of set size should be equal to the no memory condition for set sizes large then the capacity limit of 4, while the intercept should decrease. Mathematically, for set size *s*, and capacity limit *c*, the probability that a target is stored in working memory is *p* = *c*/*s*, while 1 − *p* is the probability that the target is not in working memory. The mean number of items that must be processed until the target is found is

1 $$  p+(1-p)(s + 1)/2. $$

**Fig. 1:**
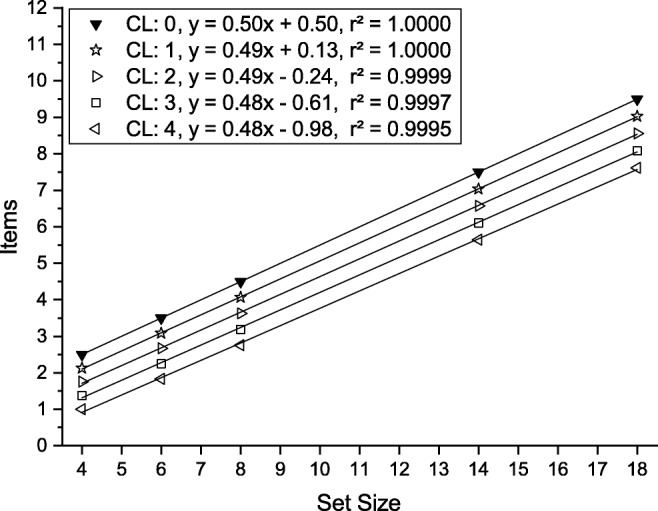
The mean number of items processed before the target is found given that a working memory of capacity CL has been filled by previewing the array. Values were computed for the same set sizes as used in the experiments (*markers*) and were regressed linearly (*lines*). See text and Eq. 1 for an explanation

Reaction time is a linear function of this number which is plotted schematically as a function of set size in Fig. 1.

**Fig. 2:**
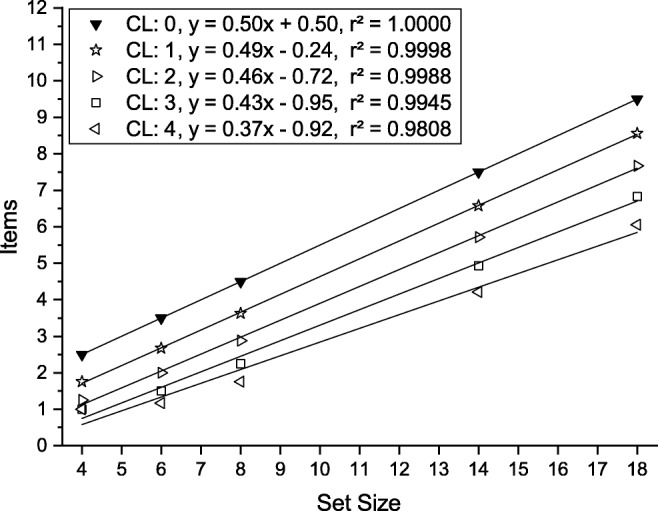
The mean number of items processed before the target is found given that a working memory of capacity CL has been filled by previewing the array and that distractor items in working memory are not examined. Values were computed for the same set sizes as used in the experiments (*markers*) and were regressed linearly (*lines*). See text and Eq. 2 for an explanation

On the other hand, if working memory was also used to prevent the attentional selection of distractor items that are in working memory, then the number of distractor items that would have to be processed would be reduced by the capacity limit, which leads to

2 $$  p + (1-p)(s - c + 1)/2, $$

a function with a shallower slope even at set sizes beyond the capacity limit illustrated schematically in Fig. 2. Such an effect would be predicted, for instance, if memory inhibited the spatial map on which attentional selection is based.

Evidence for such a form of inhibition comes from a preview search task (Watson & Humphreys, 1997) in which a subset of selected distractors is presented before the onset of the full search array. Using a dual task, Emrich et al., (2010) showed that this inhibition effect depends on the free capacity of VWM. Dube et al., (2016) were the first to successfully combine this inhibition with the guidance through a search template in VWM, in the same preview task. Whether preview of the entire scene, including the potential target, may make visual search more efficient has remained unclear, despite years of study and discussion.

The second goal that we have set ourselves for this paper is to show experimentally that working memory not only provides an overall boost to visual search by decreasing reaction time, but also increases search efficiency by spatially inhibiting locations that are in working memory. Experiment 1 establishes both in, to our knowledge, the first experimental observation of the combined effect of guidance by scene memory and inhibition from spatial working memory in a classical conjunctive search paradigm. The neural process model explains both of these roles of working memory.

Experiment 2 shows that the inhibitory effect of working memory on the efficiency of visual search can easily be disrupted, without interfering with the content and guidance from working memory, supporting the notion that inhibition comes from a separate memory subsystem. That experiment also suggests that this separate spatial memory subsystem is less stable than scene memory. The neural process model accounts for this difference as well.

## Dynamic field theory

The neural process account of visual search and its interaction with visual scene memory builds on dynamical field theory (DFT; Schöner et al., 2016), a set of mathematical concepts that captures fundamental principles of cortical organization and allows to simulate the evolution of activation patterns in populations of neurons. The activation patterns are defined over continuous feature spaces and evolve continuously in time governed by a neural dynamics. This abstracts from the discreteness of individual cells and spiking events, motivated by the dense sampling of sensorimotor spaces by broadly tuned neurons observed in cortex. Complex cognitive abilities are modeled by linking distinct populations into larger architectures through neural connections. Critically, activation patterns within populations are stabilized by lateral interaction whose strength varies as a function of distance in the underlying feature space. Functionally meaningful patterns of neural activation are thus stable states or attractors. Changes between stable states are brought about by dynamic instabilities, which allows generating autonomous sequences of the neural processing steps required for performing cognitive tasks such as visual search and memory operations.

### Neural dynamic fields

The main building block of DFT is the neural dynamic field, *u*(*x*, *t*), which evolves according to the following dynamical system:

3 $$ \begin{array}{@{}rcl@{}} \tau\dot{u}(\boldsymbol{x},t) &=& -u(\boldsymbol{x},t) +h + s(\boldsymbol{x},t) + \xi(\boldsymbol{x},t)\\ &&+ \int \omega(\boldsymbol{x}-\boldsymbol{x}^{\prime})\sigma(u(\boldsymbol{x}^{\prime},t))d\boldsymbol{x}^{\prime}. \end{array} $$

**Fig. 3:**
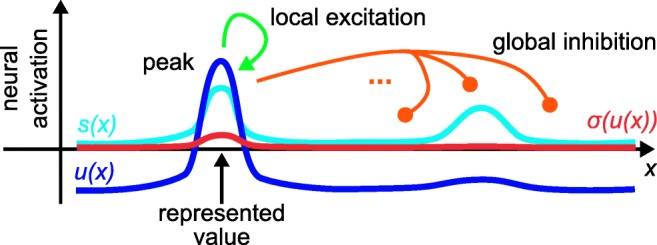
Dynamic neural field spanning a single dimension, *x*. A localized, supra-threshold peak of activation is shown together with the stabilizing local excitatory and global inhibitory interaction. The illustrated peak reflects a decision of selecting one source of localized input over another

Each field is defined over a set of dimensions, ***x***, that capture the sensory or motor parameters to which neurons in the modeled population are tuned. Which space a neural dynamic field represents is, therefore, ultimately determined by the forward connectivity from the sensory or to the motor surface. In the absence of external input, *s*(***x***, *t*), the field has a stable state at *u*(***x***, *t*) = *h* < 0, the negative resting level. Field activation above zero passes activation through the sigmoid threshold function, $\sigma (u)=1/(1+\exp [-\beta u])$, and that thresholded activation is passed on to downstream neural fields. Interaction within a neural dynamic field consists of excitatory coupling over short distances, $\boldsymbol {x}-\boldsymbol {x}^{\prime }$, and inhibitory coupling over longer distances, as modeled by the interaction kernel, $\omega (\boldsymbol {x}-\boldsymbol {x}^{\prime })$. Such coupling makes localized supra-threshold peaks of activation attractors of the neural dynamics (Fig. 3), stabilizing peaks against the influences of neural noise *ξ*(***x***, *t*), and other inputs to the field.

Supra-threshold peaks of activation are the units of representation in DFT. Each peak indicates that information about the represented space, *x*, is present and indicate through their location within that space the current perceptual estimate or motor plan. Peaks arise in the *detection instability*, when localized input pushes the activation level above threshold at that location. The sub-threshold activation pattern becomes unstable at that point. Peaks disappear in the *reverse detection instability*, when excitatory input is removed or inhibitory input arrives that pushes the peak’s activation level below threshold. Note that field activation displays hysteresis: Since local excitation becomes effective once the threshold is crossed, the input strength that will sustain an existing peak is lower than that required to create a new peak. This shields detection decisions against input fluctuations.

Higher-dimensional fields may represent the binding of feature information across different feature dimensions, e.g., color and visual location. Conversely, zero-dimensional fields are essentially neural dynamic nodes, whose dynamics

4 $$ \tau\dot{u}(t) = -u(t) +h + s(t) + c\sigma(u(t)) + \xi(t), $$

may switch from off- to on-state in the detection instability and conversely in the reverse detection instability.

Fields may be in different dynamic regimes. In the regime of *self-stabilized detection*, peaks induced by localized input are stabilized against decay and competing input. In the *selective* regime, only a single supra-threshold peak may exist stably at any moment in time. In the *sustained activation regime*, supra-threshold peaks may persist after input has been removed. Transitions between these dynamic regimes may occur in the form of instabilities, as dynamic parameters are varied such as input strengths and the resting level, *h*.

### Networks of fields/architectures

Neural fields may be coupled to other neural fields, motor systems, or sensory surfaces. Behavior and cognition emerge from such networks of fields. Because of stability of the peak solutions, fields tend to retain their dynamic regime when coupled into networks (unless they are pushed through an instability). Thus, networks of fields could be viewed as architectures. The content of each field emerges, however, only from its pattern of connectivity within the network.

Coupling among fields is directional. A field couples into another field’s dynamics (or projects onto another field), by affecting the target field’s rate of change in an additive (excitatory) or subtractive (inhibitory) manner. Only supra-threshold activation contributes to coupling, formalized by the sigmoidal threshold function, *σ*(*u*_src_), that is applied to the source field. The coupling may be modulated by a connection kernel, *c*_src,tar_(*x*, *y*), that weights how strongly locations, *x*, in the source field impact on locations, *y*, in the target field.

Target and source fields may have different dimensionality. When the source field has more dimensions than the target field, sub-spaces may be marginalized by integration. Neurally, this corresponds to a convergent or many-to-one connection scheme where connections from all field sites along the marginalized dimension in the source field converge onto a single location in the target field. When the source field has fewer dimensions, a sub-space of the target field may receive constant input (ridge or slice input) corresponding neurally to one-to-many or divergent connectivity.

### Match and mismatch detection

**Fig. 4:**
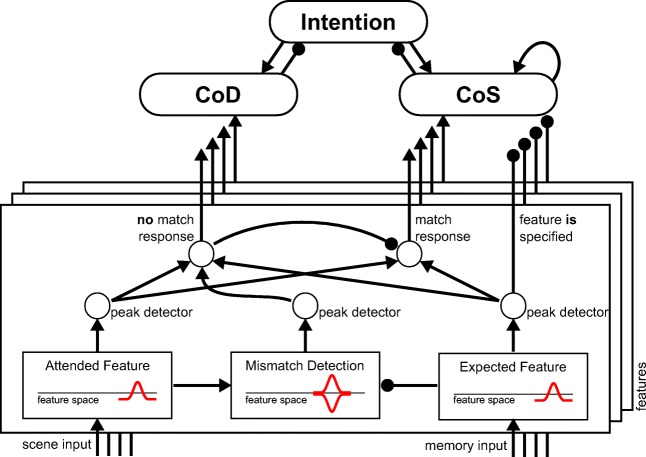
Match detection sub-network. Multidimensional feature values from two different sources are compared in parallel along each feature dimension. The mismatch detection field and connected peak detector nodes signals a mismatch if the attended, the expected, and the mismatch detection fields all carry a peak. A match is signaled if both the attended and the expected field carry peaks, but the mismatch detection field does not. A single mismatch is sufficient to activate the CoD. The CoS is activated only when a match is detected along each of the specified dimensions

A fundamental function of neural networks is to compute matches between inputs and stored representations (Rumelhart et al., 1986). In DFT, such matches engage the mechanisms of the detection instability. Specifically, a *match detection field* receives localized input from two fields such that is goes through a detection instability only if the localized inputs overlap sufficiently. The connection kernels effectively set up the metric of the match operation. Connection kernels can be designed to create a mismatch detection field that goes through the detection instability when peaks form in both input fields at non-overlapping location (Fig. 4).

### Sequences of neural processes

To generate meaningful cognitive or behavioral processes, neural dynamic networks must transition from one state to another. In neural dynamic thinking, meaningful neural representations are stable activation states that persist in the face of competition with other neural processes and may impact on down-stream neural processes to ultimately bring about behavior. The supra-threshold peaks of dynamic neural fields are stable in this sense and enables them to resist change. A prerequisite for any transition to a new state is, therefore, that the pre-transition state becomes unstable. DFT offers a general solution to this problem, the notion of a “condition of satisfaction” (CoS) (Sandamirskaya and Schöner, 2010). Any given stable neural representation pre-activates an associated inhibitory neural representation, its CoS. The pre-activation pattern reflects predictions of the conditions under which the current state has been brought to an end successfully. That is reflected in input from the sensory surface or from other parts of a neural architecture which matches the pattern of pre-activation. Upon such match, the CoS system goes through a detection instability. Its supra-threshold activation then inhibits the stable neural representation, inducing a reverse detection instability through which that state becomes deactivated. The state’s CoS is then no longer pre-activated, leading to a reverse detection instability in that field as well. At this point, the previous state and its CoS have transitioned to a sub-threshold state. Any other neural state that may have been competing with this previous activation state may now become activated through a detection instability, completing the transition to a new stable activation state.

When a stable neural representation is directly about motor behavior, predictions about its completion are predictions of direct sensory input. In many other cases, however, neural representations are about other neural representations, and predictions about the completion of such “thoughts” are predictions of the state of other neural representations. One common form of prediction is that a down-stream neural representation has created a new stable peak of activation. Signals confirming such predictions may come from *peak detectors*, neural dynamic nodes that receive input from a neural dynamic field and go through the detection instability exactly when a supra-threshold peak forms in the input field. Such nodes may be coupled in ways that bring about seemingly complex cognitive operations. For example, when they receive inputs from multiple fields, they may become activated only if peaks arise in a given number of their inputs fields.

Within neural architectures, any particular processing step may entail a whole sub-network of neural dynamic fields and nodes. Other portions of the architecture may effectively be eliminated from current processing by inhibition that is sufficiently strong to prevent the fields to generate stable peaks. Excitatory (“boosts”) or inhibitory (“deboosts”) homogeneous inputs may steer which portion of an architecture is at a given time able to generate supra-threshold activation patterns. Sometimes, such inputs are explicitly modeled by “task” nodes, which thus effectively represent a sub-network relevant to a particular task. In general, distributed patterns of activation could serve this same function. Task nodes make it simpler to explicitly address the sequential organization of different tasks through the CoS concept (see Durán et al.,, 2012, for a study of hierarchically organized sequences using this concept.)

## Neural dynamic architecture

We provide a neural dynamic processes account of three fundamental processes of visual cognition: (1) Exploring the visual array through sequences of attentional selection decisions, which each lead to the commitment of feature values at the attended locations to scene working memory; (2) attending to locations at which visual transients are detected and committing feature information from those locations to a working memory of the feature cue of visual search; (3) visually searching for locations in the visual array at which the cued feature conjunctions are detected. Both experiments and model simulations are based on the same scenario, in which participants explore a visual scene, are cued at some point to a visual search task by a sample target object that appears in the visual array, and then respond by indicating the location of a matching visual object.

**Fig. 5:**
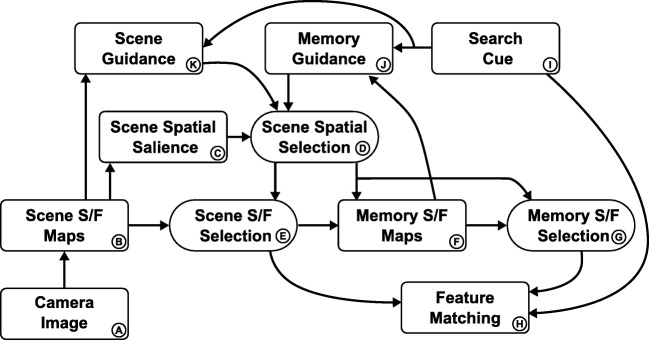
Outline of the neural dynamic architecture for visual exploration and memory formation, cue detection, and visual search. *Boxes* are neural dynamic fields or groups thereof, which are coupled as indicated by the *arrows*

Figure 5 provides an overview of the neural dynamic architecture from which these processes are generated. The boxes represent neural dynamic fields, whose coupling into a network is outlined by arrows. All neural processes evolve entirely autonomously. In other words, the model is essentially a large, but structured, system of neural integro-differential equations (of the type shown above), that evolve continuously in time driven by live visual input from a camera and by sequences of internally generated instabilities.

This architecture may look complicated, but has an inner structure that can be understood and resonates with knowledge we have about visual cognition. In the following, we first outline the structure and function of two sub-systems that play a role in all three tasks. Then we step through the three tasks of visual cognition and describe the sub-networks that bring about the required neural processes.

### Feed-forward feature and salience maps

**Fig. 6:**
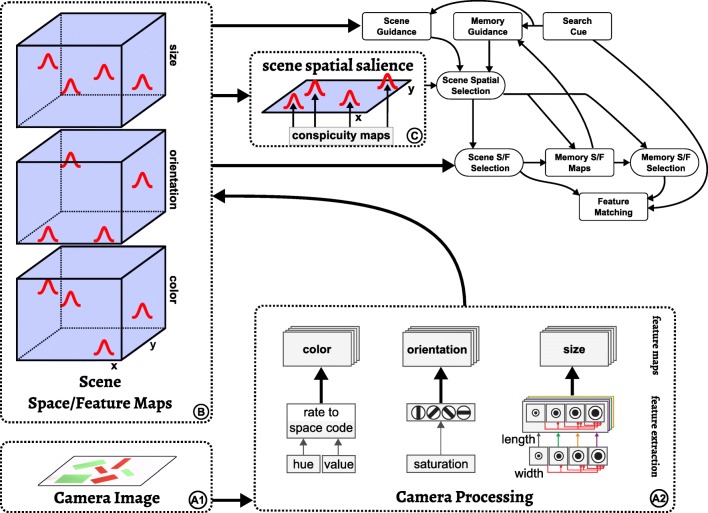
Feature extraction pathway (*left* and *bottom*) and its couplings into the remaining architecture (*top right*). See text for an explanation

Visual cognition builds on visual input from which features are extracted. This is a standard sub-task of visual cognition, that has been modeled a number of times (e.g., Itti & Koch 2000). In our particular instantiation of the sub-task, visual input may take the form of a video stream from live camera input or from sequences of synthetic images (Fig. 6). Three simple features are used in the model: *color*, *orientation* and *size* (a combination of width and length). Color is extracted by transforming RGB values into hue-space. Orientation is obtained from four elongate center-surround filters which are fed the saturation of visual input which is first passed through a threshold function. Width and length are extracted using a pyramid of center-surround filters of increasing size with a one-way inhibition along the scale dimension. The output of the feature extraction pathway provides input into three space/feature fields, which each combine two dimensions of visual space with one feature dimension (*scene space/feature maps*, *B*). These sets of three-dimensional space/feature fields will play a central role throughout the architecture. They are a mathematical formalization of Treisman’s neural feature representations.

Each of the three scene space/feature maps (B) projects to the scene spatial salience field (C), which is the sole saliency map in the architecture. These projections are purely spatial since before being applied to the scene spatial salience field the output of each space/feature map is marginalized along the feature dimension (as described earlier), thus obtaining the conspicuity map for each feature. In effect, the scene spatial salience field represents the sum of conspicuity over color, size, and orientation.

### Attentional selection

**Fig. 7:**
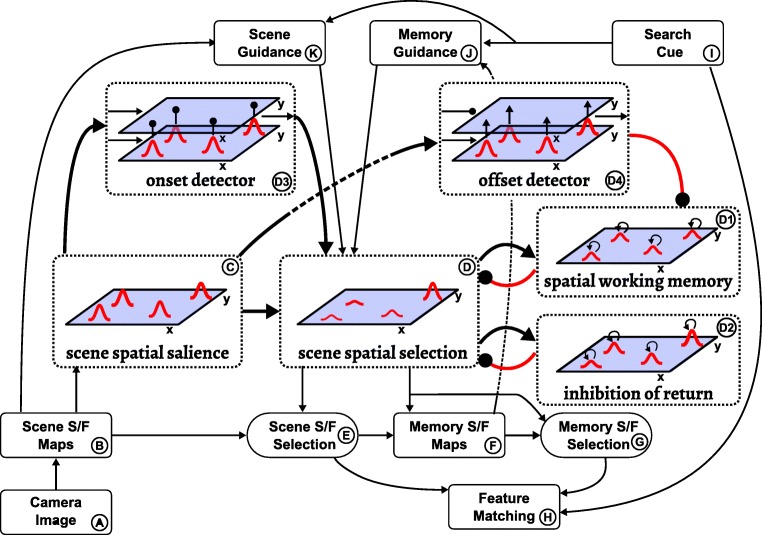
This figure highlights the attention field and its task independent inputs within the complete neural dynamic architecture

Visual cognition always entails attentional selection decisions. Figure 7 highlights the sub-system of the neural dynamic architecture that generates such selection decisions.

Central is the scene spatial selection field (*D*), which represents the current location of spatial attention. This field is in the dynamic regime of selection so that it can support only a single supra-threshold peak at any point in time. It receives multi-peak input from the salience field and selects the most salient location from among those peaks. This selection is biased by three additional sources. First, it is biased away from previously attended positions by inhibitory input from the inhibition of return memory trace (*D*2), which reflects the recent history of activation of the scene spatial selection field. Second, the first bias is supported by the self-sustained spatial working memory field (*D*1), whose representation is less stable, however, being destabilized whenever movement is detected in the scene by a two-layer offset detector (*D*4) that generates a transient activation peak when salient input peaks move or vanish. Third, attention is attracted to locations at which rapid changes of spatial salience occur, which are detected by an onset detector (*D*3). The onset detector is a two-layer neural dynamic field that generates a transient activation peak in response to tonic shifts of input (see Berger et al.,, 2012 for details).

**Fig. 8:**
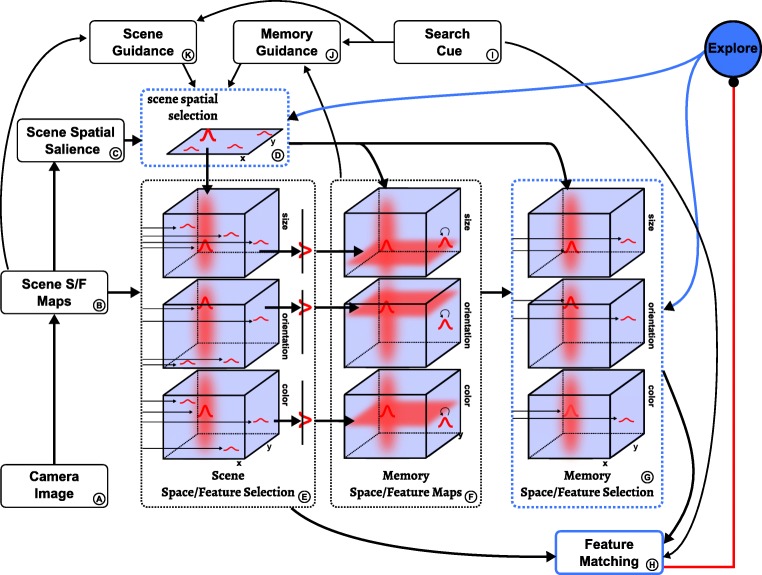
The fields involved in the exploration and memorization sub-task are highlighted within the complete neural dynamic architecture

An important role of spatial attention, represented by a self-stabilized peak in the scene spatial selection field, is to control feature binding in the manner of Treisman’s feature integration theory. Figure 8 illustrates how spatial input into a set of space/feature fields singles out the spatial locations from and to which feature values are read (as explained in the following section).

### Task 1: Visual exploration and building a working memory of the visual scene

The default behavior of the architecture is the autonomous visual exploration of the scene, during which salient locations in the visual array are attentionally selected and features at these locations are transferred to the space/ feature memory.

Figure 8 highlights the sub-network instrumental for visual exploration and memory formation. This sub-network becomes active as the “Explore” task node (top right in Fig. 8) boosts the scene spatial selection field and the memory space/feature selection fields, enabling these to generate peaks. As a consequence, the scene spatial selection field forms a peak at a single location that is favored by its inputs. The attended location provides a column-like input to a set of three-dimensional scene space/feature selection fields (*E*), which have the same structure as the scene space/feature maps described earlier (Fig. 6). Peaks form where input from the scene space/feature maps overlaps with the spatially localized columns, representing the space/feature values of the attended object. The feature information is extracted by integrating across space and provides “slice” input to another set of three-dimensional fields, the memory space/feature maps (*F*), which are in the dynamic regime of sustained activation. Where these slices overlap with column input from the scene spatial selection field, peaks form that represent the item that is being added to the scene working memory. The number of peaks that can be simultaneously sustained in the memory space/feature maps is restricted by the accumulation of inhibition as additional peaks arise. The exact number is dependent on the balance of neural inhibition and excitation in these fields and will pose a decisive factor for fitting the experimental results, as later described.

This item by item assembly of visual working memory rebinds location to feature values, just as anticipated in Treisman’s feature integration theory. One may ask why it is functionally necessary or efficient for the nervous system to first separate the initially bound space/feature information and then rebind it, requiring sequential item by item operation to avoid mismatches. Within the DFT framework, this functional need comes from the fact that the initial bound object representation is in retinal coordinates, while in visual working memory and beyond item location is represented independently of gaze. The coordinate transform that achieves this invariance is prohibitively costly if performed directly on the bound visual objects (Schneegans et al., 2016). Instead, the transformation is only performed for the spatial dimension of the fields, and the feature information is added back in as modeled here. For this paper, however, we omit coordinate transforms by assuming that all representations share the original retinal frame (i.e., that of the fixed camera), which is equivalent to assuming the absence of eye or head movements.

The memory space/feature maps provide three-dimensional input to an analogous set of three memory space/feature selection fields (*G*). In these fields, one item from the input is selected and brought above threshold, again based on overlap with column input from the scene spatial selection field. The result is an isolated representation of the memory item at the attended location. Projections from both this representation and the scene space/feature selection fields converge onto a neural feature matching mechanism (*H*, see “Match and mismatch detection”), which detects whether the attended item’s features have been successfully committed to scene working memory. When this detection occurs, the task node is deactivated through an inhibitory connection (red line in Fig. 8). This concludes one step in the exploration sequence. By default, that is, unless another task becomes active (see below), the task node is then reactivated, thus initiating another cycle of attentional selection and commitment to working memory.

### Task 2: Retaining feature cues

**Fig. 9:**
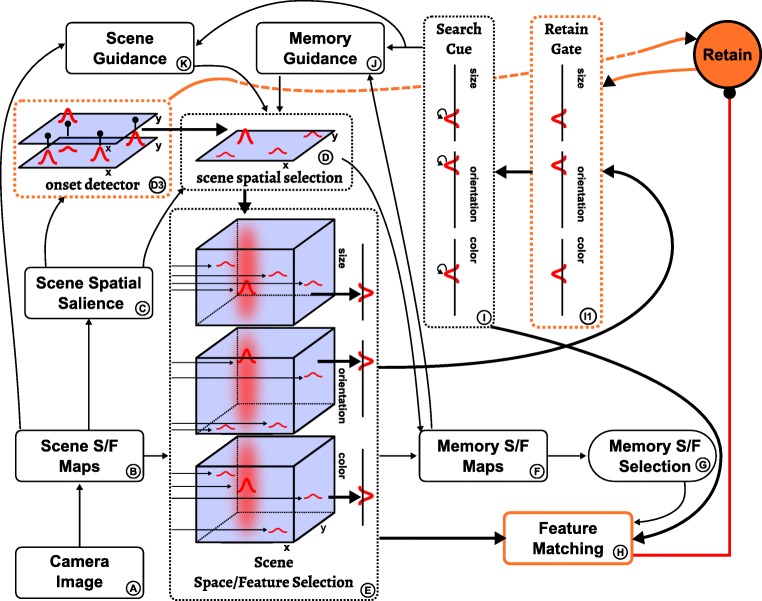
The fields engaged in the sub-task to retain feature values of a search cue are highlighted within the complete neural dynamic architecture

Figure 9 highlights the sub-network that is responsible for retaining a feature cue for visual search. It is activated by the “retain” task node, which may itself be activated from different sources depending on the cognitive task at hand. In the current context, the task node is activated by the onset detector (*D*3 in Fig. 9) when it detects a change in the visual scene.

Analogously to exploration, the retain process consists of storing currently attended feature values in self-sustained fields, the search cue fields (*I*), which are one-dimensional since only the feature values of the cue are relevant (not its position).

To forward feature values from the scene space/feature selection fields to the search cue fields, the retain node homogeneously boosts activation in the retain gate fields (*I*1), enabling them to build peaks and thus pass on activation.

The retain sub-task is terminated once the content of the search-cue fields matches the features of the currently attended item. Upon deactivation of the retain node, peaks in the attention field and the gating fields decay, whereas in the search cue fields the cue’s feature values are retained for later use.

### Task 3: Visual search for cued feature conjunctions

**Fig. 10:**
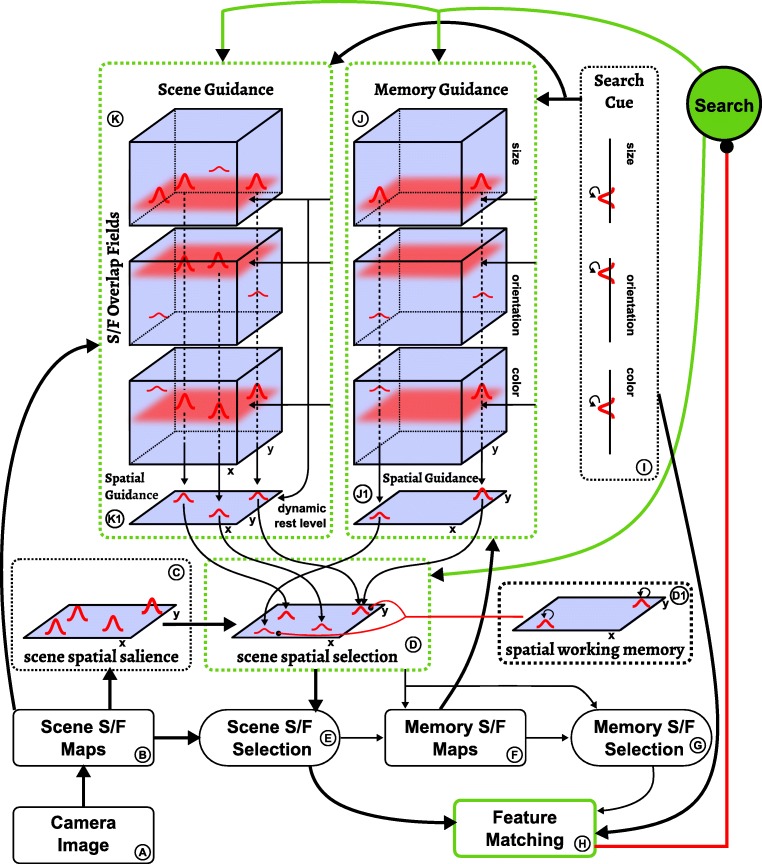
The scene and memory fields providing top-down guidance in the visual search task within the complete neural dynamic architecture

The “search” task node drives a sub-network (Fig. 10) which increases the likelihood that attention will be focused on a location where all features of the search cue are present. This is primarily achieved through top-down guidance from two sources, the scene itself (*K*) and scene memory (*J*). Each of these components includes three three-dimensional space/feature overlap fields which combine sub-threshold input from the scene maps or the memory maps, respectively, with feature input from the search cue. Supra-threshold peaks emerge at locations where there is overlap between the cued features and the scene or memory. These peaks are projected into two-dimensional spatial guidance fields (*K*1 and *J*1) from where attention in the scene spatial selection field is biased.

Importantly, the resting level of the scene spatial guidance field (*K*1) is down-regulated dynamically via inhibitory connectivity from each search cue field (*I*). The resting level thus depends on the number of cued features, decreasing as more search cue fields contain peaks. The strength of the inhibitory connections is such that when only one feature is cued it suffices for items to share only that cue feature in order to create peaks in the scene spatial guidance field (*K*1); when *n* > 1 features are cued, peaks emerge for all items that share at least *n* − 1 of the cued features. This entails that the attentional guidance is most effective in single feature search, where peaks emerge only for items that match the cue completely. It is less effective in conjunctive search, since in this case non-target items that match only *n* − 1 features of the cue become active as well. Note that this pattern emerges naturally from the requirement to down-regulate the resting level to accommodate different numbers of cued features.

The influence of memory on attentional selection described thus far is purely excitatory and based on the overlap of memory items with cue features. An additional, inhibitory influence on attentional selection comes from the spatial working memory field (*D*1), based on locations that have been committed to memory during the exploration phase. This influence decreases the likelihood that attention revisits locations that have already been visited in the exploration phase. While this may include items that match the visual search cue, the strength of inhibition is low enough to be outweighed by excitatory biases from the other sources described above. Note that the spatial working memory field is subject to the same capacity limit as the memory space/feature maps (see “??”).

The visual search process is terminated when the features at an attended location match all specified cue features. This is detected by the feature matching component (*H*), whose CoS node activates when such a match occurs, which signals task completion. If instead one or more cued feature values are not present in the attended location, the condition of dissatisfaction (CoD) node of the feature matching component becomes active and inhibits the “search” task node. This destabilizes the scene spatial selection field, which in turn leads to the CoD itself being deactivated, so that the “search” task node can reactivate and drive the attentional selection of a new location.

### Illustration of a visual search in the model

**Fig. 11:**
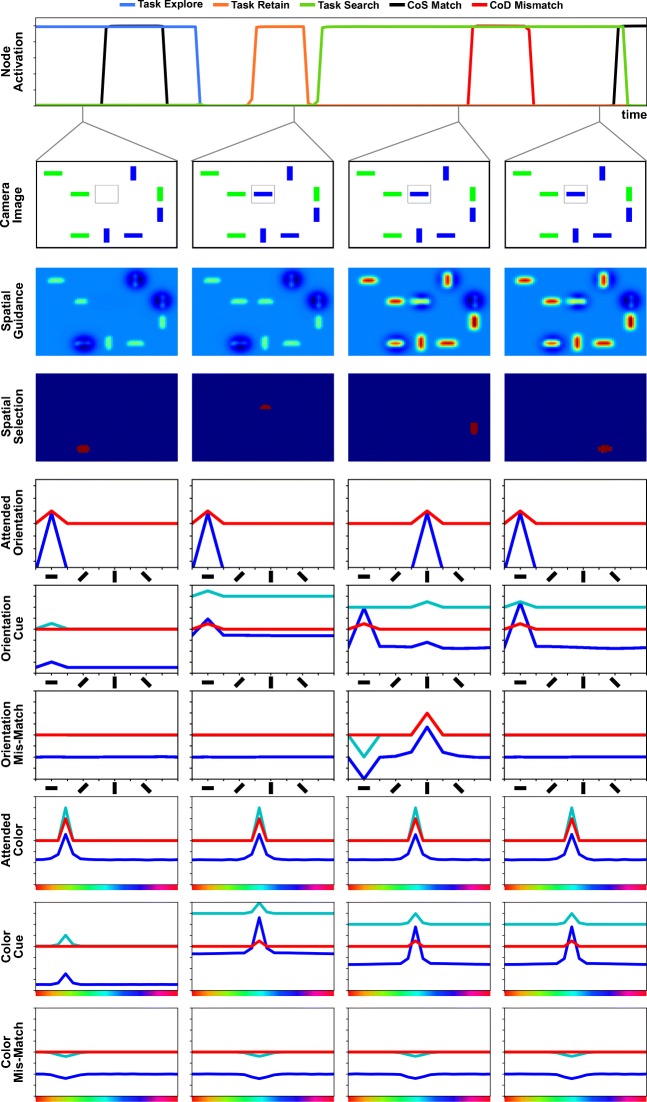
Time course of a visual search based on a cue object. The *top row* shows time courses of activation of relevant dynamic neural nodes. The rows below show activation snapshots and the visual scene at selected points in time (indicated by *grey lines*). The thresholded activation level of the combined spatial guidance (from scene and memory) and the spatial selection field is color coded (*blue indicates low*, *red indicates high levels*). The *six bottom rows* show 1D fields over orientation and color (input in *cyan*, activation in *blue*, thresholded activation in *red*)

Figure 11 demonstrates how neural events emerge from the model’s time-continuous neural dynamics that perform a conjunction search for two feature values extracted from a cue item. The task in this example is equivalent to condition 2 of Experiment 1, presented in the next section. It starts with a preview of the visual scene (first column in Fig. 11, camera image), to which the cue item is added in a next step (second column), prompting visual search for an item in the scene that has the same features as the cue (remaining columns). The task thus requires combining the two functional modes of retaining a set of cue features and visual search.

When the scene is first shown, the architecture scans it in explore mode and commits items to memory, which is the default in the absence of other task node activation. The first column of Fig. 11 shows the architecture in this mode, in a state where one object is currently in the attentional foreground (spatial selection) while the spatial guidance maps, shown as the sum of scene and memory spatial guidance fields, receive inhibition from two additional objects that have already been committed to the memory fields (not shown).

When the cue object is newly added to the visual scene (second column), the resulting transient at its location is detected by the onset detector, which serves as a trigger for the overall task, first activating the retain node. The onset detector simultaneously provides local excitation to the spatial selection field (for which only the output is shown) at the location of the transient, so that it selects the location of the cue. Feature values at that location are thus extracted and forwarded to the feature cue fields (orientation cue and color cue), where they are stored. When these fields contain peaks matching the cue features, the feature matching CoS suppresses the retain task node, which in turn allows the visual search task node to become active.

In the ensuing visual search mode (third column), the combination of scene guidance, memory guidance, and spatial working memory inhibition (not shown in Fig. 11; see *K*1, *J*1, and *D*1 in Fig. 10) biases the selection decision in the spatial selection field toward objects that share the cued features. In the scene, all but the top-right item overlap with at least *n* − 1 cue features, and thus all of these items receive a net positive bias from the guiding inputs. Together with neural noise, this leads the spatial selection field to select a non-target item that matches the color but not the orientation of the cue. This causes a peak to emerge in the orientation mismatch detection field, which in turn activates the CoD node, ultimately causing a transient deactivation of the visual search task node. This destabilizes the spatial selection field and enables the attentional selection of another item (fourth column). This time, the selected item matches the cue along all feature dimensions. In response, the CoS node of the match detection is activated, concluding the visual search task. At the end of the task, both the sought location and the associated feature values are in the attentional foreground.

## Experiment 1

The DFT model offers a concrete neural process account for the interactions between visual search and working memory, and provides the flexibility to perform different types of tasks. It can produce behavioral measures such as reaction times, which arise directly from the continuous activation dynamics in response to specific visual inputs. Here, we conduct behavioral experiments with human participants to test whether the performance of the model under different task conditions is consistent with that of human observers. We test two effects: The first is one of the most basic and well established findings in the visual search literature, namely the qualitatively different search slopes for single-feature and conjunction searches. The second is the more open question of how working memory in a preview paradigm affects the efficiency of visual search.

**Fig. 12:**
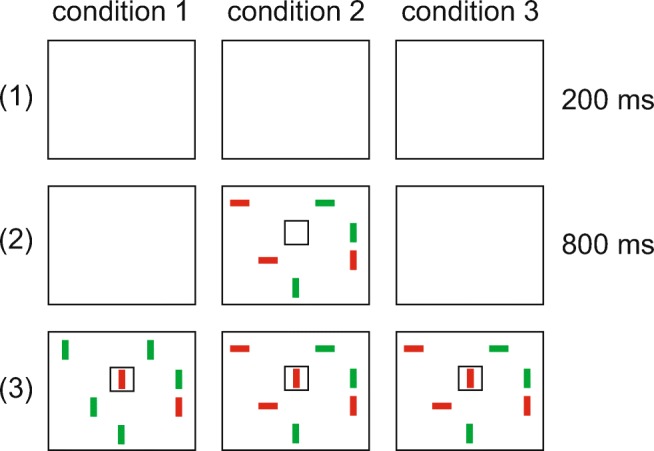
The time course of the three conditions in Experiment 1

The task in Experiment 1 was to locate an object in the visual array that exactly matches a visual cue presented in the same array. The visual array and cue were set up as single feature search (condition 1) or two-feature conjunction search (condition 2 and 3). In condition 2, the visual cue appeared 800 ms before the onset of the search array, whereas it appeared at the same time as the array in conditions 1 and 3 (Fig. 12).

### Method

#### Participants

Nineteen paid participants (nine female, ten male; age 18–27 years, mean 23.06, SD 2.51) recruited from campus completed Experiment 1. All participants reported normal or corrected-to-normal vision and normal color perception, and all except one were right-handed. All participants gave their informed consent. One was excluded from the analysis because he failed to follow the protocol.

#### Stimuli

The stimuli were organized in a 5 × 4 grid (500×400 pixels) centered on the screen, on a white background. All items were randomly positioned in a free tile (80×80 pixels) of this grid. The black-bordered middle tile (see Fig. 12) of the second row was reserved for the visual cue.

##### Condition 1: feature search

The stimulus set consisted of colored vertical bars. On each trial two colors were randomly selected from a predefined set of three colors (red, green, blue). One was defined as the target color and the other one as distractor color.

##### Condition 2 and 3: conjunction search

The stimulus set consisted of colored bars with different orientations. On each trial two colors *c*_*t*_, *c*_*d*_ and two orientations *o*_*t*_, *o*_*d*_ were randomly selected from a predefined set of three colors (red, green, blue) and four orientations (0^∘^, 45^∘^, 90^∘^, 135^∘^). Color *c*_*t*_ and orientation *o*_*t*_ were defined as the target feature conjunction. All but one distractor were assigned one of the two feature combinations *c*_*t*_ × *o*_*d*_ and *c*_*d*_ × *o*_*t*_ such that each combination was realized in an equal number of distractors. The remaining distractor was assigned the distinct feature conjunction *c*_*d*_ × *o*_*d*_. This prevented subjects from guessing the target during the search array preview phase, a strategy that would otherwise apply for small set sizes (particularly set size 4). This imposed the restriction that the number of distractors had to be odd.

#### Procedure

Each subject performed all three conditions in one consecutive session, with condition order chosen randomly. Each condition included 200 trials, for a total of 600 trials. Within each condition, five different set sizes were used (4, 6, 8, 14, and 18, each used in 40 trials). There were no target absent trials. Trial order within conditions was random.^1^

Each subject completed 30 training trials (ten for each condition) prior to the experiment. To start a trial the subject had to move the mouse pointer to a starting button below the stimulus array and click it. As shown in Fig. 12, each trial proceeded as follows: (1) empty white display for 200 ms, (2) either a preview of the search array (condition 2) or an empty white display (condition 1 and 3) for 800 ms, (3) the search array and visual cue until a response was made. Time measurement started with the onset of the visual cue (3). Participants then had to perform a speeded mouse response and click on the location of the search array item that matched the features of the visual cue. Reaction time was defined as the start of mouse movement, and location time as the time of the mouse click. If the subject moved the mouse before the onset of the visual cue or if the wrong target was clicked the trial was marked as erroneous.

Participants were instructed to locate the object in the visual array that exactly matched a visual cue which would be presented in the same array within a black-bordered square, and that such a matching item would be present in every array. They were furthermore told that the black-bordered square was not intended as a fixation point but that they could move their eyes freely^2^ and that they should start moving the mouse only once they had found the target but to then complete the movement as quickly as possible. Finally, they were informed that they did not need to click directly on the target, as any click closer to one item than to the others would be registered as selection of that item.

### Results

Trials with mouse movement prior to presentation of the search cue were excluded from analysis, as well as outliers with RT < 200 ms or RT > 6000 ms (95 trials, 0.88%). Of the remaining trials, 205 (1.92%) error trials (selections of non-target items) were removed.

#### Reaction times

**Fig. 13:**
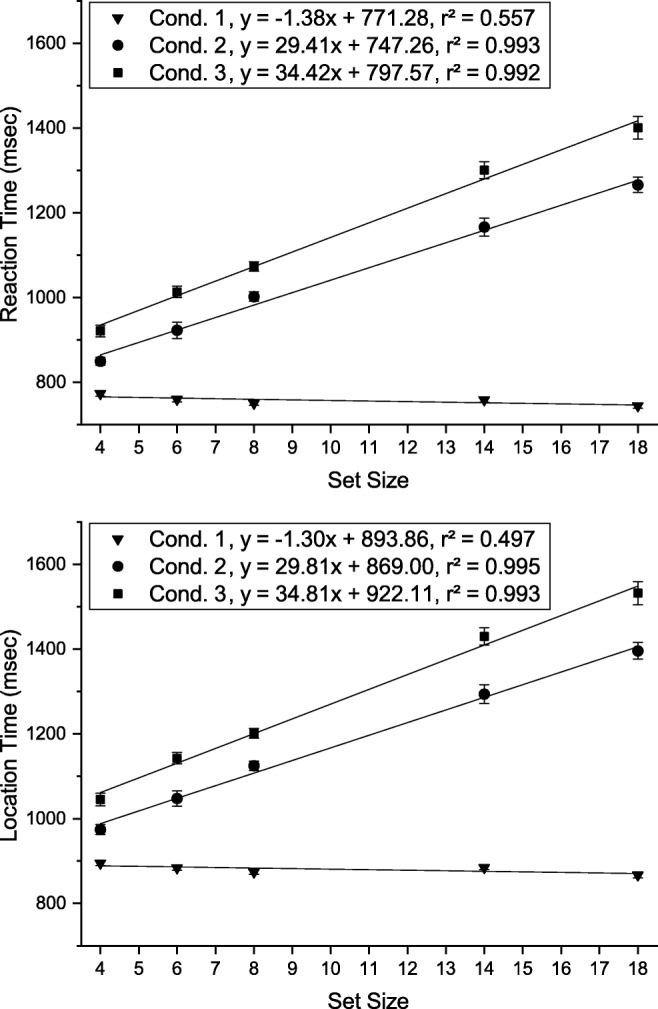
Mean reaction times (*top*) and location times (*bottom*) for the different conditions as a function of set size in Experiment 1. *Error bars* indicate ± 1 standard error of the mean (within-observer errors calculated by the method of (Cousineau, 2017))

Average reaction times and fitted slopes for each condition are depicted in Fig. 13. RTs were shortest for condition 1 and longest for condition 3.

A two-way repeated measures ANOVA of reaction times with factors *condition* (condition 1, condition 2, condition 3) and *set size* (4, 6, 8, 14, 18) revealed significant main effects of condition ($F(2,34) = 69.499, p < .001, {\eta _{p}^{2}} = .918$) and set size ($F(4,68) = 172.795, p < .001, {\eta _{p}^{2}} = .910$) as well as an interaction between them ($F(8,136) = 69.499, p < .001, {\eta _{p}^{2}} = .803$). Post hoc paired-sample *t* tests (Bonferroni adjusted *p* values) were conducted to compare the influence of condition separately for each set size. Within each set size RTs were significantly shorter in condition 1 than in condition 2 (*ps* < .001) and condition 3 (*ps* ≤ .006), and shorter in condition 2 than in condition 3 (*ps* < .05).

#### Slopes

We performed planned *t* tests on the slopes in the different conditions to determine the effects of feature vs. conjunction search and the effect of search array preview. Slopes were significantly steeper in condition 2 and condition 3 than in condition 1 (*ps* < .001). Search slopes in condition 1 were not significantly different from zero (*t*(17) = 2.462, *p* = .025, *d* = .580). Critically, we also found that the search slope in condition 3 was significantly steeper than in condition 2 (*t*(17) = 2.639, *p* = .017, *d* = .593).

#### Errors

An ANOVA on errors showed no significant effect of set size ($F(4,68)=2.082, p=.093, {\eta _{p}^{2}}=.109$), condition ($F(2,34)=2.998, p=.063, {\eta _{p}^{2}}=.150$) or their interaction ($F(8,136)=1.250, p=.275, {\eta _{p}^{2}}=.068$).

### Discussion

Condition 1 and 3 replicate the pattern of efficient single-feature search (0 ms/item) and inefficient conjunctive search (34 ms/item). Search in condition 2 was slightly more efficient (29 ms/item) than in condition 3, which is not consistent with previously reported results (Wolfe et al., 2000; Chiu and Spivey, 2012), but is in line with the calculated probabilities for a WM capacity limit of three slots as assumed in Fig. 2. The calculated expected difference of the slope (CL:3, in Fig. 2) was 14.0%, the measured difference 14.7%. These findings support the postulate that visual search is guided not only by VWM if the target was previously attended, but that SWM may as well contribute, by spatially inhibiting previously attended distractors.

#### Comparison with the model

To simulate the experiment in the DFT model, activation time courses were numerically computed using the software framework cedar (Lomp et al., 2016). The visual stimuli, the timing, and the presentation procedure were the same as in the behavioral experiment and the same number of trials was simulated. Reaction time in the model was measured as the time from initiation of the search behavior until detection of a match between the search target and a currently attended item.

To quantitatively fit model behavior to the data from Experiment 1, we adjusted the model parameters based on the behavioral data obtained in Experiment 1. Namely, both VWM and SWM of the model were tuned to have a capacity limit of four items. Note, however, that when it comes to the effect of scene preview in the context of visual search tasks this amounts to an effective capacity limit of three, due to the need to store one cue item. The same capacity limit was used in the simulations of Experiment 2.

**Fig. 14:**
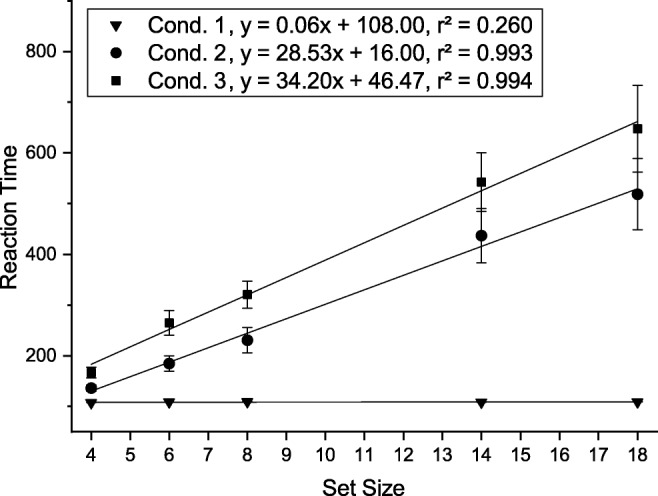
Mean reaction times for the different conditions as a function of set size produced by the model in Experiment 1. *Error bars* indicate ± 1 standard error of the mean. We note that the overall magnitude of model reaction times is scaled to ease comparison with human data, but that the relative times arise from the dynamic processes that vary with task condition and set size

Average model RTs and slopes for each condition are shown in Fig. 14. The slopes produced by the model are consistent with those measured in Experiment 1. The model reproduced both the qualitative difference between feature and conjunction searches and the quantitative effects of the search array preview. The difference of slopes between condition 2 and 3 (16.6%), however, is slightly higher than in the behavioral data (14.7%). The intercepts of the reaction time curves are significantly lower than in the behavioral experiment since the model does not capture the time needed for movement planning and execution.

## Experiment 2

**Fig. 15:**
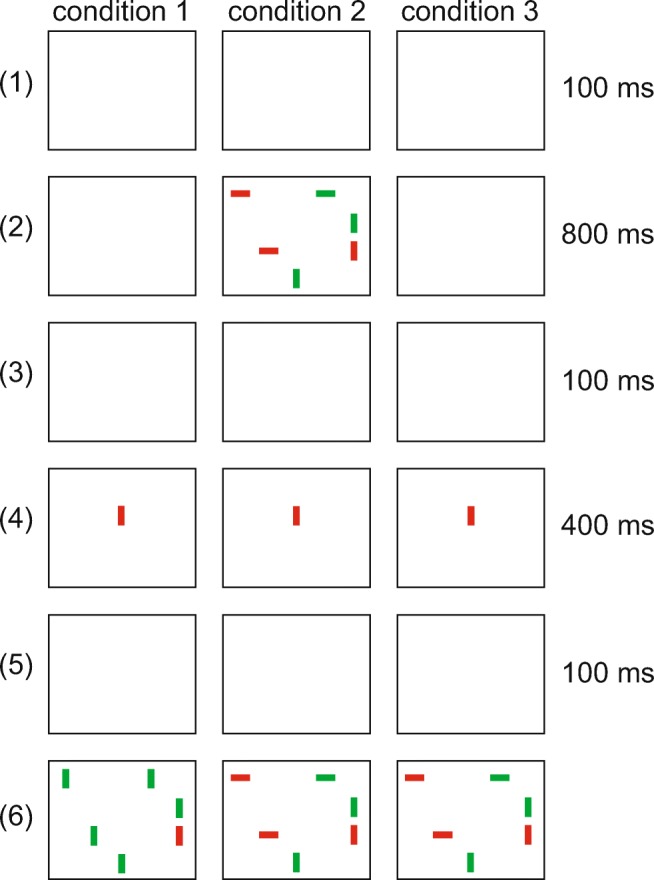
The time course of the three conditions in Experiment 2

Experiment 2 further investigated the effect of preview on search slopes and aimed to elucidate why the results of Experiment 1 contrasted with previous studies. The setup was similar to Experiment 1, again including one condition of single feature search (condition 1) and two conditions of conjunction search (condition 2 and 3). However, as Fig. 15 shows, the visual cue was presented before the search array, separated from both scene preview and search array by 100 ms of an empty white display. By this we aimed to examine the influence of intermittent presentation on the guidance and inhibition effects observed in Experiment 1. Specifically, we aimed to show a dissociation of spatial and visual working memory. We expected the visual transient induced by the 100-ms pauses to destabilize spatial working memory and thus cause the effect of inhibition to vanish. The guidance effect from the more stable visual working memory, on the other hand, was expected to be preserved. This pattern would support the notion that guidance and inhibition during visual search originate from two distinct working memory subsystems.

### Method

#### Participants

The participants were the same as in Experiment 1.

#### Stimuli

The set of stimuli was the same as in Experiment 1. Furthermore, they were organized and positioned identically to Experiment 1. The middle tile (see Fig. 15) of the second row was reserved for the visual cue and was not occupied in the subsequent search array.

#### Procedure

The procedure was identical to Experiment 1,^3^ except for changes in the time course. Each trial consisted of (1) an empty white display for 100 ms, (2) either a preview of the search array (condition 2) or an empty white display (condition 1 and 3) for 800 ms, (3) an empty white display for 100 ms, (4) the visual cue for 400 ms, (5) an empty white display for 100 ms and (6) the search array until a response was made. Time measurement started with the onset of the search array (6).

The instructions to the participants were the same as in Experiment 1.

### Results

Trials with mouse movement prior to presentation of the search cue were excluded from analysis, as well as outliers with RT < 200 ms or RT > 6000 ms (118 trials, 1.09%). Of the remaining trials, 188 (1.76%) error trials (selections of non-target items) were removed.

#### Reaction times

**Fig. 16:**
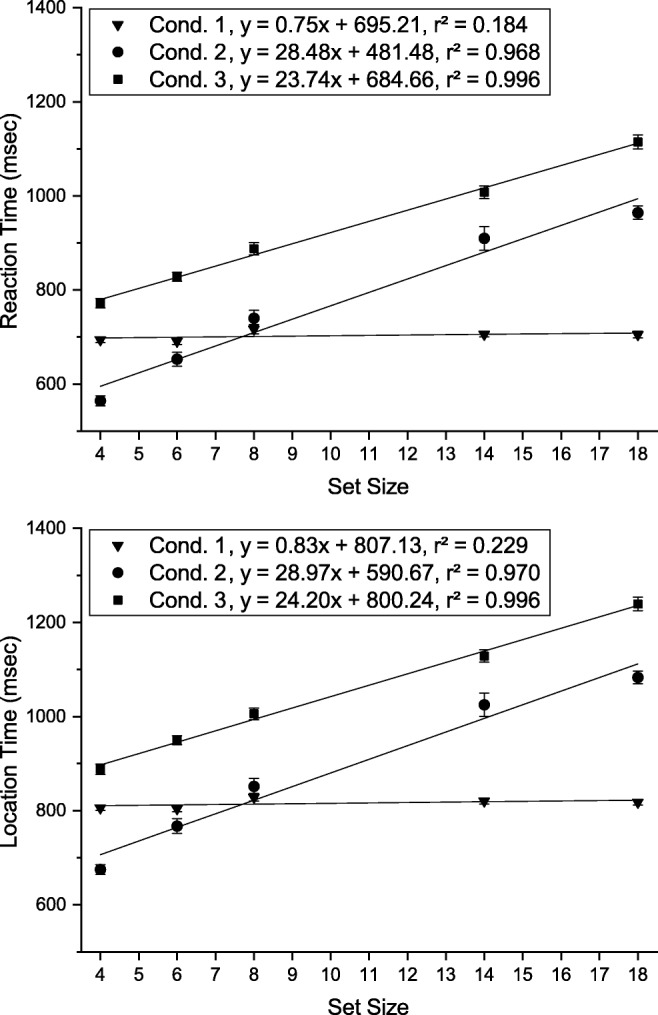
Mean reaction times (*top*) and location times (*bottom*) for the different conditions as a function of set size in Experiment 2. *Error bars* indicate ± 1 standard error of the mean (within-observer errors calculated by the method of Cousineau 2017)

Average reaction times and fitted slopes for each condition are shown in Fig. 16. A two-way repeated measures ANOVA of reaction times with factors *condition* (condition 1, condition 2, condition 3) and *set size* (4, 6, 8, 14, 18) revealed significant main effects of condition ($F(2,34) = 101.125, p < .001, {\eta _{p}^{2}} = .856$) and set size ($F(4,68) = 212.655, p < .001, {\eta _{p}^{2}} = .926$)as well as an interaction between them ($F(8,136) = 44.875, p < .001, {\eta _{p}^{2}} = .725$). Post hoc paired-sample *t* tests (Bonferroni adjusted *p* values) were conducted to compare the influence of condition separately for each set size. For all set sizes RTs were significantly shorter in condition 1 relative to condition 2 (*ps* < .001). Mean RTs were significantly shorter in condition 3 than in condition 1 for set sizes 4, 14, and 18 (*ps* < .001), but not for set sizes 6 and 8 (*ps* > 1.00). RTs were significantly shorter in condition 2 than in condition 3 for all set sizes (*ps* <= .001) with the exception of set size 14 (*p* = .060).

#### Slopes

We performed planned *t* tests on the slopes of the different conditions, and found that slopes in conditions 2 and 3 were significantly steeper than in condition 1 (*ps* < .001), reproducing the finding from Experiment 1. However, in contrast to the previous experiment, the search slope was now steeper in condition 2 (with preview of the search array) than in condition 3 (*t*(17) = 2.481, *p* = .024, *d* = .727).

We hypothesized that this effect may at least in part be explained by participants already initiating the search on memorized elements from the preview before the search array was presented again. This is suggested by the fact that, for low set sizes, mean RTs in condition 2 were even lower than in condition 1. To better estimate whether the preview in this experiment also had an effect on search slopes (based on inhibition from SWM) as seen in Experiment 1, we analyzed slopes separately for the three lowest set sizes (4, 6, 8) and the higher set sizes (8, 14, 18), assuming that the effect of memory search should be most pronounced for the low set sizes.

Slopes for set sizes 4, 6, and 8 were significantly steeper in condition 2 than in condition 3 (*t*(17) = 2.618, *p* = .036, *d* = .915), whereas there was no significant difference between the slopes of set sizes 8, 14, and 18 (*t*(17) = .283, *p* = 1.00, *d* = .087).

#### Errors

An ANOVA on errors showed a significant main effect of set size ($F(4,68)=2.981, p=.025, {\eta _{p}^{2}}=.149$), but not of condition ($F(2,34)=.163, p=.850, {\eta _{p}^{2}}=.010$). The interaction as well was non-significant ($F(8,136)=1.559, p=.143, {\eta _{p}^{2}}=.084$).

### Discussion

**Fig. 17:**
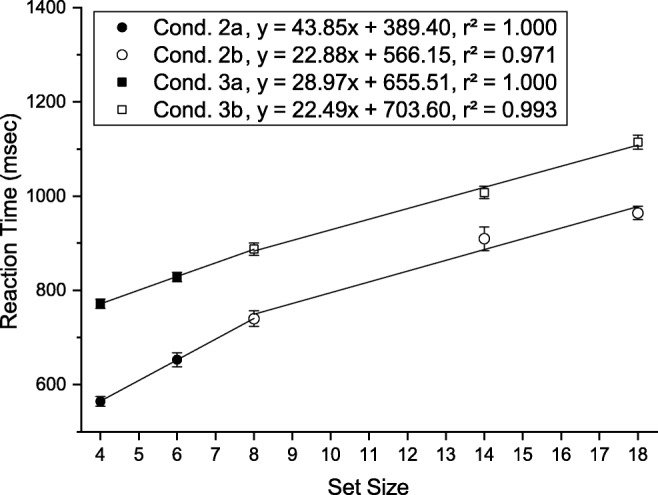
Slopes of the RTs (see Fig. 16) split into two set size intervals

Reaction time slopes in condition 1 and 3 were consistent with the pattern of results commonly seen in standard visual search tasks. The overall shorter RTs in condition 2 were consistent with the expected impact of VWM guidance. However, the intermittent presentation eliminated the inhibition effect: search efficiency did not differ between conditions 2b and 3b (see Fig. 17). Inhibition thus might be disadvantageous if the visual scene changes. The steeper slope of condition 2a (see Fig. 17) resembles the one seen for in-memory search (Kunar et al., 2008). Together with the fact that RTs were very short in comparison to condition 1 (see Fig. 16) this hints that subjects started in-memory search as soon as they had processed the visual cue and thus before the actual onset of the search array. Since this strategy is constrained by the capacity limit of visual working memory it probably works best for set sizes below 8, explaining why the slope between set sizes 4 and 8 was steeper than for higher set sizes. Finally, that RTs were overall shorter than in Experiment 1 likely reflects that the visual cue in Experiment 2 could be processed before the onset of the search array.

#### Comparison with the model

**Fig. 18:**
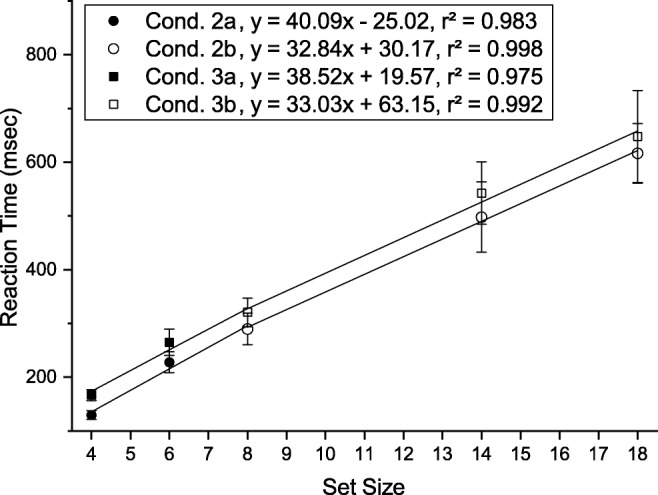
Mean reaction times for the different conditions as a function of set size produced by the model in Experiment 2. *Error bars* indicate ± 1 standard error of the mean. The results of condition 3 comes from Fig. 14. For better comparability we used the same starting point of measurement as in Experiment 1. As in Fig. 14, the overall magnitude of model reaction times was scaled for comparison with human data

We simulated condition 2 of Experiment 2 by supplying the model with a sequence of visual inputs according to the presentation order in that condition. Model parameters were identical to those for simulations of Experiment 1. The resulting model RT means and fitted slopes are shown in Fig. 18, along with those from the simulation of condition 3 of Experiment 1 (see Fig. 14). Performance in conditions 1 and 3 of Experiment 2 would be identical to conditions 1 and 3 in Experiment 1, so that we did not run these simulations again and used the results from the previous simulation.

For set sizes 8, 14, and 18, the difference of slopes between the two conditions is consistent with the slope difference observed in Experiment 2 (both near zero), thus showing no inhibition effect. As concerns the model, this results from the reset of SWM when a visual transient is induced by the disappearance of the preview array. The model also replicates the slightly steeper slopes over set sizes 4, 6, and 8 seen in condition 3 of Experiment 2. It does not, however, capture the slope of condition 2 for these lower set sizes. This is due to the fact that, even though the array is partly memorized, the model does not perform pure in-memory search in the absence of a visual scene, because search mode in the model is triggered only in the presence of a visual scene.

## General discussion

We have presented an account of interactions between visual working memory and visual search using a combined approach of computational modeling and behavioral experiments. Our first goal in this study was to provide a neural process model of visual search that accounts for established findings in this field (for reviews, see Carrasco (2011), Wolfe and Horowitz 2017) but additionally incorporates a mechanism for scene working memory. This allows us to explore possible interactions between these two systems in a biologically plausible model. The behavioral literature over the past two decades has clearly established that working memory influences visual search in various ways, but many details of their interactions are still controversial (for reviews, see Hollingworth, 2012a, Donk, 2006; Olivers et al.,, 2006).

The model we propose employs various mechanisms of visual processing that have been established in previous work, and brings them together into a fully integrated neural-dynamic architecture implemented in the framework of DFT. The feedforward path of the model is closely related to the saliency map model (Itti & Koch, 2000), a standard model of visual attention and visual search that realizes key aspects of feature integration theory (Treisman & Gelade, 1980). We modeled color, orientation, and size as basic visual features, since these have been shown to be effective in guiding visual search (Wolfe & Horowitz, 2017).

Our model is consistent with key aspects of guided search (Wolfe, 2007), in that it employs top-down guidance of visual attention by a featural cue (see also Hamker 2005), for an earlier neural-dynamic implementation of this mechanism). Since guidance depends on the metric differences between target and distractors (Duncan & Humphrey, 1989; Friedman-Hill & Wolfe, 1995; Wolfe, 1998), our model proposes a simple normalization mechanism of neural activation, which is based on the number of cued features and therefore scales naturally for higher feature conjunctions (Nordfang & Wolfe, 2014). This mechanism also produces the qualitative differences between single-feature and conjunction search in the model.

A key feature of the DFT model is that it performs a sequential processing of the visual scene, selecting individual items through spatial attention (comparable to the attentional bottleneck proposed in guided search). This sequential process is realized as an integral part of the neural dynamics, and emerges from transitions between different stabilized states within the neural populations without any algorithmic control structures outside of the neural model. Consistent with Treisman’s feature integration theory (Treisman & Gelade, 1980), the sequential attentional selection is required in the model to bind the different visual features of an object together and compare them to a template in conjunctive search tasks.

The same sequential process is also employed in the model to form a multi-item and multi-feature scene memory, in a mechanism adapted from Schneegans et al., (2016). This working memory mechanism can in this respect be viewed as a neural implementation of Treisman’s object file theory (Kahneman et al., 1992). However, instead of invoking the information processing concept of object files, the working memory representations are here realized as feature maps bound via their shared spatial dimensions, with sustained activation peaks as working memory states (Wei et al., 2012; Johnson et al., 2014). The concept of binding via space employed here is supported by patterns of binding errors in behavioral experiments (Treisman and Zhang, 2006; Schneegans & Bays, 2017).

This means that visual search and visual working memory systems in the DFT model share the same representational format, the same processing mechanism, and to some extent the same model components. This allows a high degree of integration, such that visual search can be extended naturally from the currently viewed scene to a scene held in memory. A substantial overlap between mechanisms for visual search in present visual scenes and in visual working memory is supported by behavioral, fMRI and MEG studies (Griffin & Nobre, 2003; Kuo et al.,, 2009, 2016), which show that the same mechanisms of spatial selection operate in both scenarios. Moreover, visual search also interacts with long-term memory, such that detailed visual information is incidentally acquired during visual search (Castelhano & Henderson, 2005; Williams et al., 2005), and associations of distractor configuration with target location (or distractor identities with target identity) can be learned over a few trials and used to facilitate subsequent searches (Chun & Jiang, 1998, 2003; Jiang & Leung, 2005).

A prominent paradigm to test the effects of working memory on visual search performance is the preview paradigm. Hollingworth (2009) showed the benefits of a scene preview in a natural setting, with a guidance effect both for a preview without the search target, as well as an additional guidance effect from memory for object-location bindings if the target was present. Hillstrom et al., (2012) extended this work by showing that scene gist information (from a briefly glimpsed preview) can improve search efficiency. However, this effect was not found for randomly ordered search arrays, indicating that it is specific to naturalistic scenes.

A common finding in the preview paradigm is that mean RTs are reduced if a preview of the search array is provided. Becker and Pashler (2005) argued that this is strong evidence for guidance of attention by VWM (but see Kunar et al.,(2008) for an opposing opinion). However, the slope of the search curve (reaction time over different set sizes) has not been found to be decreased by the preview in these experiments, which has led to the conclusion that search efficiency is unaffected by the preview benefit.

We chose the scene preview paradigm as key behavioral task to address with the DFT model, since it allows for a formation of scene memory that is unbiased by the specific search task. Within this paradigm, we specifically addressed the question why the preview benefits observed for natural scenes by Hollingworth (2009) and Hillstrom et al., (2012) did not generalize to randomly arranged search arrays. It is plausible, of course, that knowledge of the scene layout conveys specific benefits if this layout is meaningful (as in a natural scene) rather than arbitrary (as in a random search array). But a random search array should be equally memorized during the preview (within the given capacity limits of working memory). Assuming that search for items already held in working memory can be completed almost instantaneously, this predicts that RTs should be substantially reduced if a preview is provided. This has indeed been found (Becker and Pashler, 2005), and the reduction of search time is consistent with previously postulated working memory capacity limits of about four items (taking into account that the search target itself is also held in VWM, see Woodman et al.,^2007^).

However, the question remains why the preview did not lead to an increase of search efficiency as reflected in the slope of the search curve, which should be observable if the locations of all objects already held in scene memory are de-prioritized during the search process.

Such an effect is implemented in the DFT model through inhibition from spatial working memory (SWM) to the spatial selection field used in visual search.

We attempted to find this predicted effect in the experimental part of our study, and in Experiment 1 we demonstrated for the first time an increase of search efficiency arising from a preview of a random search array. The search slopes and the preview benefit were well accounted for by simulation of the behavioral task in the DFT model. In Experiment 2, we tested the robustness of this effect, and found that a small change in stimulus presentation settings (with preview and search target separated by blank screens) caused the efficiency benefit to disappear. This may explain why the effect has not been observed in previous studies (Wolfe et al., 2000; Chiu & Spivey, 2012).

To account for the different results from the two experiments in the DFT model, we propose that the robust effect of overall RT benefits and the more fragile effect of inhibition of memorized locations arise from two different memory sub-systems. We attribute the former to guidance from VWM, and the latter to inhibition from a dedicated SWM representation that stores the locations of previously attended objects. This distinction between VWM and SWM is supported by various studies (for reviews, see Baddeley & Logie 1999; Smith & Jonides, 1997). Of particular relevance here is the observation that visual search is impaired by a concurrent spatial working memory task (Woodman & Luck, 2004), while no such impairment was found from a concurrent object working memory task (Woodman et al., 2001).

To explain the different results in the preview conditions of Experiment 1 and Experiment 2, we further assume that the SWM field is inhibited and the memory representation thereby resets when the current visual array disappears. This seems desirable to prepare for the presentation of new visual input, as otherwise new objects that appear at previously occupied locations would be treated as already inspected. As a result, SWM of the preview affects visual search in Experiment 1 (where the preview/search array remains continuously visible), but not in Experiment 2 (where preview and search array are separated by a blank screen and the presentation of the search target). We hypothesize that a similar effect may also occur in the experiment of Chiu and Spivey (2012) and account for their failure to find an increase in search efficiency with a preview. Here, the search target is presented outside of the search array, and processing it will require a transient shift of attention that may likewise disrupt or reset the SWM representation. We note that the experiment in Chiu and Spivey (2012) also differed in other factors from the previous study, such as the size of the search cue and the occurrence of target-absent trials. We cannot rule out that any of these factors also contributed to the different results.

Accounting for the integration of visual search with scene memory is one step toward capturing visual search under more naturalistic conditions (Hollingworth, 2012a). In the real world, humans know much more about an object they are looking for than a few feature values. In particular, they make use of an object’s category (Yang & Zelinsky, 2009). How such categorical information may be used to guide visual search is not yet well understood. One idea is that templates representing object classes may be matched to current visual input (Heinke & Humphreys, 2003; Heinke & Backhaus, 2011; Lomp et al., 2017; Abadi et al., 2019). Doing this at least partially in parallel across the visual array requires non-trivial neural operations in which a mapping from all visual locations to a spatial representation of the template is narrowed down during the recognition process to a selected location. In deep neural networks, parallel search is achieved trivially by weight sharing, in which the neural connectivity relevant to recognition is “copied” to every location in the visual array, clearly not a neurally plausible idea. An alternative might be that every category is learned together with a few salient visual feature values so that visual search based on these simple features could be used in a first step, followed by more complex object recognition at the attentionally selected spatial location.

### Conclusions

We presented a first neural process account of feature integration theory that avoids any element of information processing while modeling a complete visual search paradigm, including the detection of the search cue from visual transients, its commitment to feature memory, the autonomous generation of a sequence of attentional selection decisions, and the matching of the cued feature values and the feature values extracted at each attended location. The model accounts for conjunctive searches in a way that is consistent with the original notion of binding through space. The model also autonomously explores the visual array and builds a scene working memory, which we have used in earlier work (Schneegans et al., 2016) to account for the signatures of feature integration theory in change detection tasks.

The model is based on the principle of neural dynamics, so that all processing steps emerge from time- and state-continuous neural processes. Such models must satisfy a large number of constraints, not all of which are represented by quantitative experimental data. For instance, the capacity of the model to proceed autonomously from one processing step to another makes demands on the conditions under which particular instabilities take place, that strongly constrain the range of possible model parameters. For models of this nature, the true number of “free” parameters is thus not easy to estimate. In a certain sense, such a model is a proof of principle that does not preclude that, in principle, other models within the same framework could provide similar or better fit to experimental data. In practice, however, it is quite difficult to build even a single neural dynamic model that is consistent with all functional constraints. Existence proofs of this nature are thus actually quite valuable.

To provide and test specific predictions that help move the model beyond an existence proof, we explored in depth the interaction between visual search and working memory. We discovered that allowing observers to first build a scene working memory not only speeds visual search as often reported, but also increases search efficiency, an effect that has remained elusive for a long time. In our neural model, two separate neural pathways bring about the two effects of working memory. Working memory speeds search through the guidance mechanism in which the item is immediately found if it is in working memory. The increase of efficiency comes from inhibition of locations that are already in spatial working memory and are then no longer examined during search. Because that spatial working memory is fragile, so is the enhanced search efficiency, as we demonstrated in a second experiment. This may explain past difficulties to establish this effect.

## Footnotes

1 The trial order within conditions for the first five subjects were ordered by ascending set size due to technical issues. A repeated measures ANOVA with factors *condition*, *set size* and the between-subjects factor *random order* showed that the missing randomness of trials had no impact on the results (no effect on condition ($F(2,32) = .231, p = .795, {\eta _{p}^{2}} = .014$), set size ($F(4,64) = .902, p = .468, {\eta _{p}^{2}} = .053$) or the interaction between them ($F(8,128) = .592, p = .783, {\eta _{p}^{2}} = .036$)), so that we included these participants in all other analysis.

2 Al-Aidroos et al., (2012) found evidence (see Exp. 2B) that the effect of preview inhibition could only be measured if the subjects were allowed to freely move their eyes.

3 As in Experiment 1 the trial order within conditions for the first five subjects were ordered by ascending set size due to technical issues. A repeated measures ANOVA with factors *condition*, *set size* and the between-subjects factor *random order* showed that the missing randomness of trials had no impact on the results (no effect on condition ($F(2,32) = .584, p = .564, {\eta _{p}^{2}} = .035$), set size ($F(4,64) = .245, p = .912, {\eta _{p}^{2}} = .015$) or the interaction between them ($F(8,128) = .843, p = .567, {\eta _{p}^{2}} = .050$)), so that we included these participants in all other analysis.

## References

[CR1] Abadi AK, Yahya K, Amini M, Friston K, Heinke D (2019). Excitatory versus inhibitory feedback in Bayesian formulations of scene construction. Journal of the Royal Society Interface.

[CR2] Al-Aidroos N, Emrich SM, Ferber S, Pratt J (2012). Visual working memory supports the inhibition of previously processed information: Evidence from preview search. Journal of Experimental Psychology: Human Perception and Performance.

[CR3] Anderson EJ, Mannan S, Rees G, Sumner P, Kennard C (2010). Overlapping functional anatomy for working memory and visual search. Experimental Brain Research.

[CR4] Baddeley A, Logie R (1999). Working memory: The multiple-component model.

[CR5] Becker MW, Pashler H (2005). Awareness of the continuously visible: Information acquisition during preview. Perception & Psychophysics.

[CR6] Berger, M, Faubel, C, Norman, J, Hock, H, & Schöner, G. (2012). *The counter-change model of motion perception: An account based on dynamic field theory*, vol 7552 LNCS. Springer.

[CR7] Bundesen C (1990). A theory of visual attention. Psychological Review.

[CR8] Carrasco M (2011). Visual attention: The past 25 years. Vision Research.

[CR9] Castelhano MS, Henderson JM (2005). Incidental visual memory for objects in scenes. Visual Cognition.

[CR10] Chikkerur S, Serre T, Tan C, Poggio T (2010). What and where: a Bayesian inference theory of attention. Vision Research.

[CR11] Chiu, E, & Spivey, M (2012). The role of preview and incremental delivery on visual search. In: *Proceedings of the Annual Meeting of the Cognitive Science Society* (pp. 34).

[CR12] Chun MM, Jiang Y (1998). Contextual cueing: Implicit learning and memory of visual context guides spatial attention. Cognitive Psychology.

[CR13] Chun MM, Jiang Y (2003). Implicit, long-term spatial contextual memory. Journal of Experimental Psychology: Learning, Memory, and Cognition.

[CR14] Cousineau D (2017). Varieties of confidence intervals. Advances in Cognitive Psychology.

[CR15] Deco G, Rolls ET (2004). A neurodynamical cortical model of visual attention and invariant object recognition. Vision Research.

[CR16] Donk M (2006). The preview benefit: Visual marking, feature-based inhibition, temporal segregation, or onset capture?. Visual Cognition.

[CR17] Dube B, Basciano A, Emrich SM, Al-Aidroos N (2016). Visual working memory simultaneously guides facilitation and inhibition during visual search. Attention, Perception, & Psychophysics.

[CR18] Duncan J, Humphrey GW (1989). Visual search and stimulus similarity. Psychological Review.

[CR19] Durán, B., Sandamirskaya, Y., & Schöner, G. (2012). A dynamic field architecture for the generation of hierarchically organized sequences. In A. Villa, W. Duch, P. Érdi, F. Masulli, & G. Palm (Eds.) *Artificial Neural Networks and Machine Learning – ICANN 2012, Lecture Notes in Computer Science LNCS*, (Vol. 7552 pp. 25–32).

[CR20] Emrich SM, Al-Aidroos N, Pratt J, Ferber S (2010). Finding memory in search: The effect of visual working memory load on visual search. The Quarterly Journal of Experimental Psychology.

[CR21] Erlhagen, W, Bastian, A, Jancke, D, Riehle, A, & Schöner, G (1999). The distribution of neuronal population activation (DPA) as a tool to study interaction and integration in cortical representations. *Journal of Neuroscience Methods, 94*(1). 10.1016/s0165-0270(99)00125-9 10638815

[CR22] Fix J, Rougier N, Alexandre F (2011). A dynamic neural field approach to the covert and overt deployment of spatial attention. Cognitive Computation.

[CR23] Folk, C L (2015). Controlling spatial attention: Lessons from the lab and implications for everyday life. In J.M. Fawcett, E.F. Risko, & A. Kingstone (Eds.) *The Handbook of Attention* (pp. 3–25): The MIT Press / Bradford Books.

[CR24] Friedman-Hill S, Wolfe JM (1995). Second-order parallel processing: Visual search for the odd item in a subset. Journal of Experimental Psychology: Human Perception and Performance.

[CR25] Griffin IC, Nobre AC (2003). Orienting attention to locations in internal representations. Journal of Cognitive Neuroscience.

[CR26] Hamker FH (2005). The emergence of attention by population-based inference and its role in distributed processing and cognitive control of vision. Computer Vision and Image Understanding.

[CR27] Hamker FH (2006). Modeling feature-based attention as an active top-down inference process. BioSystems.

[CR28] Heinke D, Humphreys GW (2003). Attention, spatial representation, and visual neglect: Simulating emergent attention and spatial memory in the selective attention for identification model (SAIM). Psychological Review.

[CR29] Heinke D, Backhaus A (2011). Modelling visual search with the selective attention for identification model (vs-SAIM): A novel explanation for visual search asymmetries. Cognitive Computation.

[CR30] Hillstrom AP, Scholey H, Liversedge SP, Benson V (2012). The effect of the first glimpse at a scene on eye movements during search. Psychonomic Bulletin & Review.

[CR31] Hollingworth A (2009). Two forms of scene memory guide visual search: Memory for scene context and memory for the binding of target object to scene location. Visual Cognition.

[CR32] Hollingworth, A. (2012a). Guidance of visual search by memory and knowledge. In *The influence of attention, learning, and motivation on visual search* (pp. 63–8): Springer. 10.1007/978-1-4614-4794-8_4 PMC3875155 23437630

[CR33] Hollingworth A (2012). Task specificity and the influence of memory on visual search: Comment on võ and Wolfe (2012). Journal of Experimental Psychology: Human Perception and Performance.

[CR34] Humphreys GW, Müller HJ (1993). Search via recursive rejection (SERR): A connectionist model of visual search. Cognitive Psychology.

[CR35] Humphreys GW (2016). Feature confirmation in object perception: Feature integration theory 26 years on from the Treisman Bartlett lecture. Quarterly Journal of Experimental Psychology.

[CR36] Itti L, Koch C (2000). A saliency-based search mechanism for overt and covert shifts of visual attention. Vision Research.

[CR37] Jiang Y, Leung AW (2005). Implicit learning of ignored visual context. Psychonomic Bulletin & Review.

[CR38] Johnson JS, Simmering VR, Buss AT (2014). Beyond slots and resources: Grounding cognitive concepts in neural dynamics. Attention, Perception, & Psychophysics.

[CR39] Kahneman D, Treisman AM, Gibbs BJ (1992). The reviewing of object files: Object-specific integration of information. Cognitive Psychology.

[CR40] Kunar MA, Flusberg S, Wolfe JM (2008). The role of memory and restricted context in repeated visual search. Perception & Psychophysics.

[CR41] Kuo BC, Rao A, Lepsien J, Nobre AC (2009). Searching for targets within the spatial layout of visual short-term memory. Journal of Neuroscience.

[CR42] Kuo BC, Nobre AC, Scerif G, Astle DE (2016). Top-down activation of spatiotopic sensory codes in perceptual and working memory search. Journal of Cognitive Neuroscience.

[CR43] Lomp, O, Richter, M, Zibner, SKU, & Schöner, G. (2016). Developing dynamic field theory architectures for embodied cognitive systems with cedar. *Frontiers in Neurorobotics, 10*,14. 10.3389/fnbot.2016.00014 PMC5089998 27853431

[CR44] Lomp, O, Faubel, C, & Schöner, G. (2017). A neural-dynamic architecture for concurrent estimation of object pose and identity. *Frontiers in Neurorobotics, 11*,23. 10.3389/fnbot.2017.00023 PMC5408094 28503145

[CR45] Luck, SJ, & Vogel, EK (1997). The capacity of visual working memory for features and conjunctions. *Nature, 390*, 279 = 281. 10.1038/36846 9384378

[CR46] Ma WJ, Husain M, Bays PM (2014). Changing concepts of working memory. Nature Neuroscience.

[CR47] Moran, R, Zehetleitner, M, Muller, HJ, & Usher, M (2013). Competitive guided search: Meeting the challenge of benchmark RT distributions. *Journal of Vision, 13*(8), 24–24. 10.1167/13.8.24 23887047

[CR48] Nordfang M, Wolfe JM (2014). Guided search for triple conjunctions. Attention, Perception, & Psychophysics.

[CR49] Olivers CN, Humphreys GW, Braithwaite JJ (2006). The preview search task: Evidence for visual marking. Visual Cognition.

[CR50] Purushothaman, G, & Bradley, DC (2005). Neural population code for fine perceptual decisions in area MT. *Nature Neuroscience, 8*(1), 99–106. 10.1038/nn1373 15608633

[CR51] Richter M, Lins J, Schöner G (2017). A neural dynamic model generates descriptions of object-oriented actions. Topics in Cognitive Science.

[CR52] Rumelhart, DE, McClelland JL, & The PDP Research Group (Eds.) (1986). *Parallel distributed Processing–Volume 1: Foundations*. Cambridge: The MIT Press–A Bradford Book.

[CR53] Sandamirskaya Y, Schöner G (2010). An embodied account of serial order: How instabilities drive sequence generation. Neural Networks.

[CR54] Schneegans, S, Spencer, J P, & Schȯner, G. (2016). Integrating ‘what’ and ‘where’: Visual working memory for objects in a scene, In Schöner, G., Spencer, J.P., DFT Research Group, T. (Eds.) Dynamic thinking: A primer on dynamic field theory: Oxford University Press, Chap 8.

[CR55] Schneegans, S, & Bays, P M (2017). Neural architecture for feature binding in visual working memory. *Journal of Neuroscience*, 3493–16. 10.1523/JNEUROSCI.3493-16.2017 PMC5394900 28270569

[CR56] Schöner, G., Spencer, J P, & DFT Research group, T. (2016). Dynamic thinking: A primer on dynamic field theory: Oxford University Press.

[CR57] Smith EE, Jonides J (1997). Working memory: a view from neuroimaging. Cognitive Psychology.

[CR58] Soto D, Heinke D, Humphreys GW, Blanco MJ (2005). Early, involuntary top-down guidance of attention from working memory. Journal of Experimental Psychology: Human Perception and Performance.

[CR59] Soto D, Hodsoll J, Rotshtein P, Humphreys GW (2008). Automatic guidance of attention from working memory. Trends in Cognitive Sciences.

[CR60] Tatler, B W, & Land, MF (2016). Everyday Visual Attention. In Kingstone, A., Fawcett, J.M., Risko, E.F. (Eds.) *The Handbook of Attention*: The MIT Press, Chap 17.

[CR61] Treisman, AM (1998). Feature binding, attention and object perception. *Philosophical Transactions of the Royal Society (London) B Biological Sciences, 353*, 1295–1306. 10.1098/rstb.1998.0284 PMC1692340 9770223

[CR62] Treisman AM, Gelade G (1980). A feature-integration theory of attention. Cognitive Psychology.

[CR63] Treisman, AM, & Zhang, W (2006). Location and Binding in Visual Working Memory. *Memory & Cognition, 34*(8), 1704– 1719. 10.3758/bf03195932 PMC1868390 17489296

[CR64] Võ MLH, Henderson JM (2010). The time course of initial scene processing for eye movement guidance in natural scene search. Journal of Vision.

[CR65] Võ MLH, Wolfe JM (2012). When does repeated search in scenes involve memory? Looking at versus looking for objects in scenes. Journal of Experimental Psychology: Human Perception and Performance.

[CR66] Võ MLH, Wolfe JM (2015). The role of memory for visual search in scenes. Annals of the New York Academy of Sciences.

[CR67] Watson DG, Humphreys GW (1997). Visual marking: prioritizing selection for new objects by top-down attentional inhibition of old objects. Psychological Review.

[CR68] Wei Z, Wang XJ, Wang DH (2012). From distributed resources to limited slots in multiple-item working memory: a spiking network model with normalization. Journal of Neuroscience.

[CR69] Wheeler ME, Treisman AM (2002). Binding in short-term visual memory. Journal of Experimental Psychology: General.

[CR70] Williams CC, Henderson JM (2005). Incidental visual memory for targets and distractors in visual search. Perception & Psychophysics.

[CR71] Wolfe JM (1998). What can 1 million trials tell us about visual search?. Psychological Science.

[CR72] Wolfe JM, Klempen N, Dahlen K (2000). Postattentive vision. Journal of Experimental Psychology: Human Perception and Performance.

[CR73] Wolfe JM, Oliva A, Butcher SJ, Arsenio HC (2002). An unbinding problem? The disintegration of visible, previously attended objects does not attract attention. Journal of Vision.

[CR74] Wolfe, J.M. (2007). Guided Search 4.0: Current Progress with a Model of Visual Search. In Gray, W.D. (Ed.) *Integrated Models of Cognitive Systems* (pp. 99–119): Oxford University Press, chap 8.

[CR75] Wolfe, JM (2015). Visual Search. In Kingstone, A., Fawcett, J.M., Risko, E.F. (Eds.) *The Handbook of Attention, chap 2* (pp. 27–56): The MIT Press.

[CR76] Wolfe JM, Horowitz TS (2017). Five factors that guide attention in visual search. Nature Human Behaviour.

[CR77] Wolfe, J M (2018). Visual search. In Wixted, J.T. (Ed.) *Stevens’ handbook of experimental psychology and cognitive neuroscience, Developmental and Social Psychology*: Wiley.

[CR78] Woodman GF, Vogel EK, Luck SJ (2001). Visual search remains efficient when visual working memory is full. Psychological Science.

[CR79] Woodman GF, Luck SJ (2004). Visual search is slowed when visuospatial working memory is occupied. Psychonomic Bulletin & Review.

[CR80] Woodman GF, Luck SJ, Schall JD (2007). The role of working memory representations in the control of attention. Cerebral Cortex.

[CR81] Yang, H, & Zelinsky, GJ (2009). Visual search is guided to categorically-defined targets. *Vision Research, 49*(16), 2095–2103. 10.1016/j.visres.2009.05.017 PMC2756560 19500615

